# Profiling Cell Signaling Networks at Single-cell Resolution*

**DOI:** 10.1074/mcp.R119.001790

**Published:** 2020-03-04

**Authors:** Xiao-Kang Lun, Bernd Bodenmiller

**Affiliations:** ‡ Institute of Molecular Life Sciences, University of Zürich, 8057 Zürich, Switzerland; § Molecular Life Sciences PhD Program, Life Science Zürich Graduate School, ETH Zürich and University of Zürich, 8057 Zürich, Switzerland

**Keywords:** Signaling circuits, phosphoproteome, systems biology, assay development, pathway analysis, single-cell analysis

## Abstract

Signaling networks process intra- and extracellular information to modulate the functions of a cell. Deregulation of signaling networks results in abnormal cellular physiological states and often drives diseases. Network responses to a stimulus or a drug treatment can be highly heterogeneous across cells in a tissue because of many sources of cellular genetic and non-genetic variance. Signaling network heterogeneity is the key to many biological processes, such as cell differentiation and drug resistance. Only recently, the emergence of multiplexed single-cell measurement technologies has made it possible to evaluate this heterogeneity. In this review, we categorize currently established single-cell signaling network profiling approaches by their methodology, coverage, and application, and we discuss the advantages and limitations of each type of technology. We also describe the available computational tools for network characterization using single-cell data and discuss potential confounding factors that need to be considered in single-cell signaling network analyses.

## Abstract (precis)

Signaling network responses can be highly heterogeneous across cells in a tissue because of many sources of genetic and non-genetic variance. The emergence of multiplexed single-cell technologies has made it possible to evaluate this heterogeneity. In this review, we categorize currently established single-cell signaling network profiling approaches by their methodology, coverage, and application, and we discuss the advantages and limitations of these technologies. We describe the computational tools for network characterization using single-cell data and discuss potential confounding factors that may affect single-cell analyses.

## Graphical Abstract

**Figure:**
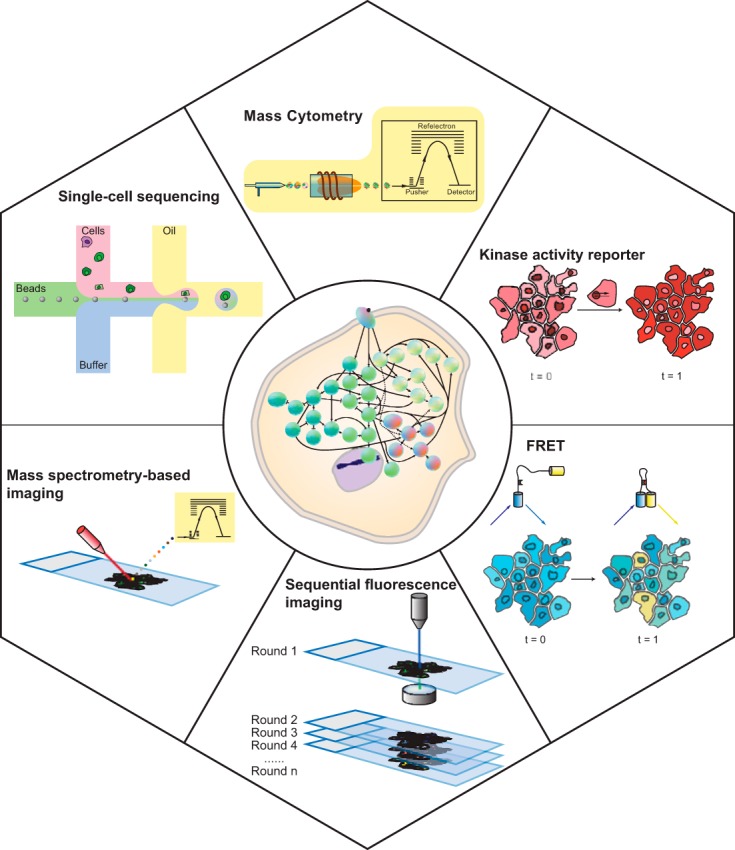

**Highlights**

- Signaling networks can be highly heterogeneous across cells in a tissue.
- Various technologies allow analyzing signaling networks at single-cell resolution.
- The advantages and limitations of each single-cell approach are summarized.
- Confounding factors in single-cell signaling network analysis are discussed.

## Cell-to-Cell Heterogeneity in the Signal Transduction Response

Signaling pathways mediate cell communication and coordinate cellular functions such as proliferation, differentiation, and energy metabolism (1–4). They are often regulated by phosphorylation events mediated by kinases and phosphatases that result in the controlled activity of downstream effector molecules. Early research in signal transduction focused on delineating individual signaling pathways (or cassettes of signaling events) and understanding the enzyme-substrate relationships within these pathways. For example, after the discovery that MAP kinases (mitogen-activated protein kinases) are serine/threonine kinases regulated by phosphotyrosine signaling, identification of both upstream kinases and downstream targets enabled the definition of “cascades” of specific phosphorylation events that respond to distinct cues for each MAP family member (5–7).

Although this reductionist view of signaling events helped understand key principles of signal relay, it soon became apparent that signaling pathways are rarely independent within cells and living organisms, but are instead integrated. In general, crosstalk between two signaling pathways produces an output that differs from which would be triggered by only one of the pathways, and involves direct or indirect connections between the pathways (8, 9). For example, an enzyme in one pathway may directly phosphorylate and regulate a component of another pathway. Alternatively, indirect crosstalk can, for example, involve the transcriptional output of one pathway controlling the expression of components of another pathway (8). Together with positive and negative feedback loops within pathways, these crosstalks can fine-tune or amplify signal in a context-dependent manner, resulting in a composite output (10).

**Fig. 1.**
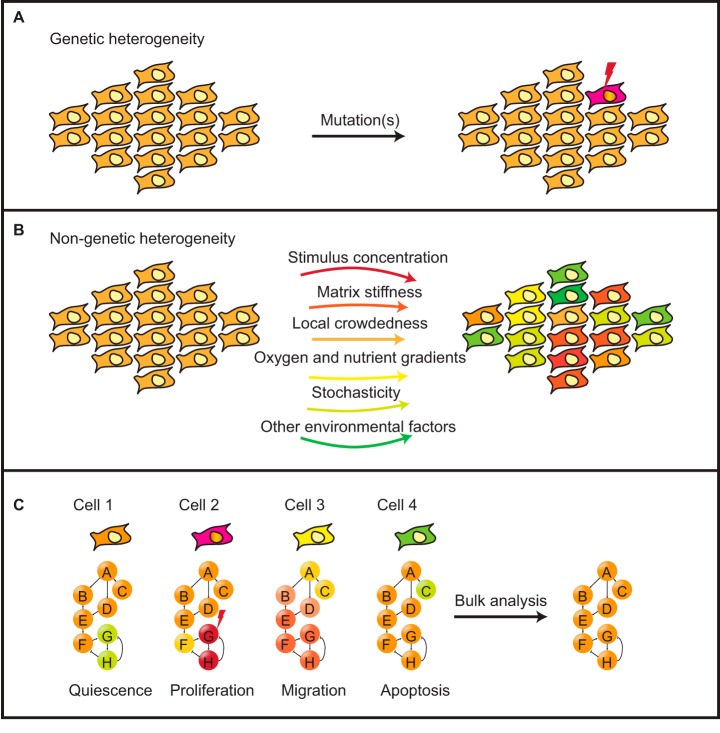
**Signaling network heterogeneity in cell populations.** *A*, Mutated signaling proteins (e.g., kinases) may cause genetic heterogeneity in a population of cells and leads to differential signaling networks. *B*, Non-genetic signaling network heterogeneity may origin from extrinsic factors including stimulus concentration, matrix stiffness, local crowdedness, oxygen and nutrient gradients, as well as the intrinsic noise. *C*, Signaling network heterogeneity results in phenotypical variances in a population of cells. Bulk analysis averages these variances, resulting in misinterpretation of cell signaling network behaviors and cell phenotypes.

Although they remain essential to the understanding of signaling, bulk biochemical studies of pathways and networks, for example using global phosphoproteomics profiling (11–13), also do not account for cell-to-cell variability. At the single-cell level, network responses can be highly variable depending on cell type and on environmental conditions. These differences are because of many sources of genetic and non-genetic heterogeneity in individual cells (Fig. 1).

Genetic heterogeneity is often explained by mutations that affect the present and functionality of proteins and causes inherited phenotypical variability in a population of cells (Fig. 1*A*). Mutated signaling proteins may reshape signaling network structures and result in different response dynamics (14). In cancer, genomic instability leads to the accumulation of mutations that results in discrete genetic abnormities that further the signaling response heterogeneity in cells from the same tumor (15).

Non-genetic heterogeneity is known as differential phenotypes in cells sharing the identical genome. Causes of non-genetic heterogeneity include epigenetic regulation and so-called intrinsic and extrinsic factors. Intrinsic heterogeneity denotes the inherent stochasticity of biomolecules present in a cell (16) that may affect chemical processes involved in the life cycles of an mRNA or a protein. Extrinsic heterogeneity is generated by factors that modulate the transcriptome and the proteome of cells in an ununiformed manner. Variables such as concentration of a stimulus (identities of neighboring cells), extracellular matrix stiffness (17), local crowdedness and spatial constraints (18, 19), nutrient and oxygen gradients (20), cell cycle and cell volume (21) integratively provide heterogeneous signaling network responses in isogenic cells (Fig. 1*B*).

The signaling network heterogeneity resulting from these factors is crucial for biological processes, such as cell differentiation and tissue development (22) and the maintenance of a functional bio-system (23). During the progression of many diseases, including cancer, the signaling network heterogeneity also causes an increase in phenotypical complexity that may reduce the efficacy of therapeutic interventions (24). For decades, these single-cell-level variances could not be systematically profiled because of technical limitations (Fig. 1*C*). Our understanding of signaling network behaviors in diseases is therefore often incomplete. The recent emergence of multiplexed single-cell measurement technologies has made it possible to profile signaling networks cell-by-cell. This allows to uncover the origin of genetic or non-genetic heterogeneity (25, 26), to analyze the variation of signaling networks affected by this heterogeneity (27, 28), and to evaluate downstream transcriptional and phenotypic effects induced by modulation of a signaling pathway or network (29, 30).

## The Single-cell Era of Signaling Network Analysis

### Single-cell Analysis with High Multiplexity

**Fig. 2.**
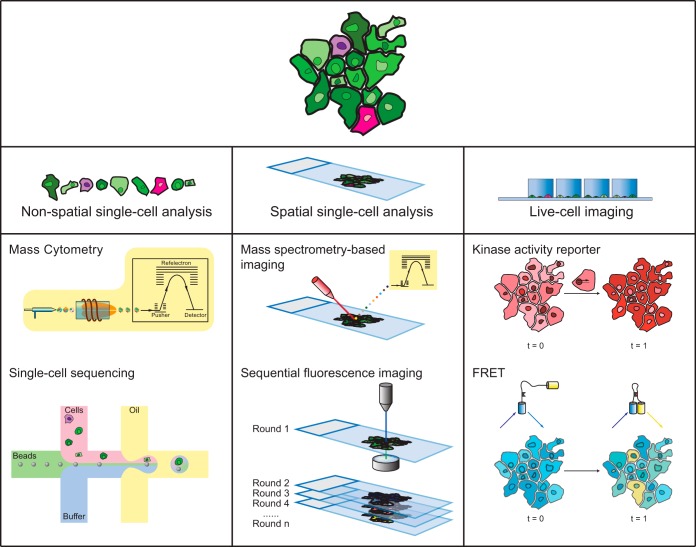
**Approaches to analyze cell signaling networks at single-cell resolution.** Information on signaling network states in individual cells can be analyzed in cell suspension with mass cytometry, which allows simultaneous measurement of about 50 markers such as phosphorylation levels of signaling proteins and markers of cell phenotype. Single-cell RNA sequencing technologies allow transcriptomics profiling that can be used to infer cell signaling states. Multiplexed cell signaling profiling can be performed *in situ* with mass spectrometry-based imaging methods or with sequential immuno-based fluorescence imaging; these methods preserve spatial information. Live-cell imaging methods (e.g., kinase translocation reporters, FRET) can be used to monitor dynamic signaling behaviors in real time with single-cell resolution, although with lower multiplexing capability.

Single-cell analysis has been performed since the invention of the microscope. Conventional microscopic methods are used to visualize cell structure, assess protein expression levels, and study cellular and subcellular spatial properties. These studies are facilitated by genetic or immunological fluorescent protein tagging methods (31). Because of the limited multiplexing capacity of conventional microscopy, independent experiments using different batches of cells are generally required, resulting in the loss of relationships between assessed markers. This makes it challenging to study signaling mechanisms at the network level using conventional microscopy. The era of 'omics has made it possible to simultaneously measure transcriptomic and proteomic information (32). Protein phosphorylation, one of the most critical post-translational modifications for signaling transduction, can be globally analyzed with phosphoproteomic approaches (33). The lack of sensitivity makes it challenging to apply these methods to single-cell measurements, however. Very recently, novel approaches that enable multiplexed antibody detection capacity (34–39) and signal amplification (40–42) have made it possible to explore cellular phosphorylation landscapes and signaling regulatory network structure cell-by-cell in heterogeneous samples. Here we summarize currently available approaches for signaling network analysis at single-cell resolution (Fig. 2 and Table I).

**Table I:** Comparison of single-cell approaches for signaling network analysis

Technique	Multiplicity	Through put	Cost	Sample type	Target of measurement	Spatial resolution	Sensitivity
Flow cytometry	Up to 30	Very high	Low	Single cells stained with fluorophore-conjugated antibodies	Proteins and protein modifications. High number of additional assays available.	N/A	High
Mass cytometry	Up to 50	High	Low	Single cells stained with metal isotope-conjugated antibodies	Proteins, protein modifications and transcripts	N/A	High
Single-cell immuno-sequencing (CITE-seq and REAP-seq, etc.)	Unlimited	Medium	High	Single cells stained with DNA oligonucleotide-labeled antibodies	Proteins and protein modifications	N/A	Medium
Lab-on-chip and microfluidics (SCBC and scWesterns)	10	Medium	Low	Single-cell lysis	Proteins and protein modifications	N/A	High
Single-cell proteomics	Unlimited	Very low	High	Single-cell lysis	Proteins and protein modifications	N/A	Low
Single-cell RNA-seq	Unlimited	Medium	High	Single-cell lysis	mRNA	N/A	Medium
Multiplexed imaging based on sequential antibody staining (MELC, MxIF, CycIF, 4i, etc.)	Up to 90	Medium	Low	Fixed cell or tissue slides	Proteins and protein modifications	High	High
Multiplexed imaging based on sequential antibody detection (immune-SABER and CODEX, etc.)	30	High	Low	Fixed cell or tissue slides	Proteins and protein modifications	High	High
Imaging mass cytometry (IMC)	Up to 50	Medium	Medium	Fixed cell or tissue slides	Proteins and protein modifications	Medium	Medium
Multiplexed ion beam imaging (MIBI)	Up to 50	Medium	High	Fixed cell or tissue slides	Proteins and protein modifications	High	Medium
MALDI-based imaging	Unlimited	Medium	High	Fixed tissue slides	Lipids and metabolites	Low	Low
*In situ* sequencing (FISSEQ)	Unlimited	Low	High	Fixed cell or tissue slides	mRNA	High	Low
Fluorescence *in situ* hybridization (MERFISH and seqFISH, etc.)	100s	Low	Low	Fixed cell or tissue slides	Genomic DNA and mRNA	High	High
Kinase translocation reporter	3	Medium	Low	Live cells	Kinases	High	High
FRET	Up to 6	Medium	Low	Live cells	Kinases or interactive proteins	High	High

### Non-spatial Single-Cell Analysis Based on Immunological Approaches

#### 

##### Flow Cytometry

Flow cytometry uses fluorophore-labeled antibodies to detect and quantify protein abundance in individual cells. It has been used to monitor relationships between multiple phosphorylation sites and correlations between phosphorylation states, functional readouts, and lineage-specific markers in complex populations of cells (43). With the capability to simultaneously measure ∼10 (up to 30 in more advanced setups) phosphoproteins and phospholipids, flow cytometry-based single-cell analysis has recently been combined with inhibitor perturbation assays enabling the inference of signaling circuits and the reconstruction of signaling networks (44). The development of fluorescent cell barcoding has greatly increased the throughput of flow cytometry-based intracellular signaling analysis. It is now routinely implemented as a screening tool to quantify cellular responses to kinase inhibitors in individual cell types in heterogeneous populations (45, 46). However, because of the overlap of the fluorescent spectra of the fluorescent dyes used to label antibodies, the number of markers that can be analyzed simultaneously by flow cytometry remains limited, and signaling networks can only be sparsely or partially interrogated using this technique. Nevertheless, with the advantages of throughput and accessibility, flow cytometry is one of the most used methods for single-cell signaling assessments in research and diagnosis (47, 48).

##### Mass Cytometry

Mass cytometry is based on inductively coupled plasma time-of-flight mass spectrometry and a single-cell sample introduction system (34). In mass cytometry, metal isotope-tagged antibodies are used to label proteins or protein modifications in cells. Metal tags allow multiplicity considerably higher than possible with flow cytometry. During the mass cytometry measurement, each stained single cell is vaporized, atomized, and ionized. The metals in the formed ion cloud are quantitatively analyzed by the mass spectrometer to yield a high-dimensional single-cell proteomic readout (Fig. 2, left panel) (34, 49). A mass cytometry analysis simultaneously quantifies up to 50 cell-surface or intracellular markers, including phosphorylation sites, with high analytical throughput of around 500 cells per second and millions of events per sample. A mass-tag barcoding strategy allows simultaneous measurement of hundreds of samples, eliminating batch effects that confound conventional measurements and reducing the workload (27, 50, 51).

The mass cytometry does not have sensitivity superior to flow cytometry, but cell auto-fluorescence, which interferes with quantification of a fluorescently labeled marker in flow cytometry, is not an issue with mass cytometry (34). Although minor spill-over between channels of the mass cytometer occurs because of metal impurity, mass overlap, and oxidation (52), these events are manageable with proper experimental design and can be removed computationally (53).

Mass cytometry has been used in drug screening (50). Relationships between all pairs of measured phosphorylation sites can be computed to infer network responses to a stimulus (54) or to trace the network reshaping through a phenotypical transition (55). When coupled to a transient overexpression technique, mass cytometry-based signaling profiling enables assessment of how intracellular signaling states and dynamics depend on protein abundance. These types of experiments have revealed novel signaling mechanisms involved in cancer progression and drug resistance (27, 56).

##### Single-cell Immuno-sequencing

As only about 50 metal isotopes are routinely used in mass cytometry, deep profiling of phosphoprotein networks is not possible. Two recently developed techniques, REAP-seq and CITE-seq, barcode antibodies with oligonucleotides to increase multiplexing. These methods allow detection of targeted proteins by single-cell sequencing simultaneously with quantification of RNA transcriptomes in the same cells (57, 58). More than 10 million distinct barcodes can be generated with a 12-mer oligonucleotide (4^12^), making the measurable parameters in this type of methods virtually unlimited. REAP-seq and CITE-seq have been implemented for cell-surface marker staining, and it is expected that these techniques will soon be used at the intracellular level for comprehensive single-cell signal profiling. Yet, sequencing-based approaches suffer from high technical variance and are therefore less quantitative than flow and mass cytometry methods. Experimental cycles are also slower in sequencing methods compared with flow and mass cytometry, making optimizations more time-consuming.

##### Lab-on-Chip and Microfluidics

Lab-on-chip technologies, such as single-cell barcode chips (SCBCs) and single-cell Western blotting (scWesterns), are more sensitive than cytometric methods and allow detection of low-abundance proteins (59–61). These approaches have been applied to resolve single-cell signaling network variations and functional heterogeneity (60, 61). Investigations of single-cell signaling kinetics can also be performed using microfluidic systems that allow fine time resolution and accurate dose control of the profiled stimulus (62).

### Non-spatial Single-cell Analysis Based on 'Omics Approaches

Immunostaining-based techniques allow multi-dimensional deep profiling of signaling networks at single-cell resolution, but also face three main limitations: First, the selection of measured targets is based on prior knowledge, so these methods are not suitable for exploratory studies. Second, not all targets of interest are measurable because of the high dependence on antibody availability. Third, given different antigen-binding affinities, quantifications cannot be compared across antibodies. Fortunately, the development of several antibody-free 'omics approaches has provided complementary techniques that do not suffer from these limitations.

#### 

##### Single-cell Proteomics by Mass Spectrometry

A big challenge for single-cell mass spectrometry is the comparably low sensitivity of the technique, especially for low abundance proteins, which is because of sample loss during processing, the absence of amplification approaches for proteins, and limitations to instrument sensitivity. Advances in sample processing and alternative strategies to overcome these limitations have been introduced in the past few years. For instance, SCoPE-MS (Single Cell ProtEomics by mass spectrometry) uses labeling with tandem mass tags to embed single mammalian cells in hundreds of carrier cells to separate the identification (from multiple “carrier” cells) from quantification of proteins in single cells, enabling quantification of over 1000 proteins per single cell (63). A second-generation version of this method SCoPE2 that includes optimized sample preparation steps was used to assess over 2,000 proteins in 356 single cells within 85 h (64). This field of research is very active, and further advances in this type of approach and their adaptation to profile the phosphoproteome in single cells are expected to help push the boundaries of single-cell proteomics. Nevertheless, the low throughput and high cost are likely to remain significant limitations.

##### Single-cell Transcriptomics and Epigenomics

Single-cell sequencing techniques (40, 65) do not directly measure protein abundance and cannot detect functional protein modifications that reflect signaling network activation. However, with the strength to quantify global RNA expression and identify whole-genome transcriptional regulation landscapes, these approaches can be used to infer transcriptional regulatory networks and the dynamics of signaling pathways in response to a stimulus (Fig. 2, left panel). For example, single-cell RNA-seq revealed a paracrine signaling-required repression of the inflammatory program (66). Single-cell epigenomes can now be measured with ATAC-seq, which sequences transposase-accessible chromatin (67, 68). By coupling single-cell transcriptomics and epigenomics analyses, the network of transcriptional regulation during stem cell differentiation was profiled, and crucial signaling pathways during the transition from quiescence to proliferation and differentiation were identified (69, 70).

### Spatial Single-cell Analysis Based on Immunological Approaches

Spatial variables (*e.g.* cell contacts and protein localizations) might act as crucial determinants during the processing of cellular signaling information. These properties cannot be assessed with the single-cell analytical methods described above as cell detachment or tissue dissociation is required for sample acquisition. Imaging-based cytometry and 'omics techniques can preserve cellular spatial information and are also capable of resolving subcellular details of protein localization. The additional spatial dimension gained with these approaches provides clues to sources of cellular heterogeneity and facilitates the profiling of signaling network behaviors.

#### 

##### Sequential Fluorescence Imaging

Spatial information on protein localization and tissue organization can be acquired through fluorescence microscopic measurements of cell monolayers or tissue sections. Fluorescence spectrum overlap limits the number of channels that can be detected in a simultaneous measurement, however. To achieve the high multiplicity required for signaling network profiling, technologies have been developed that allow sequential imaging of the same specimen without influencing antigen abundance or tissue structure (Fig. 2, middle panel). The first generation of sequential imaging approaches applies fluorophore-labeled antibodies to detect targets of interest (37, 38, 71, 72). Specifically, MELC implements photo-bleaching after each round of antibody staining and imaging cycle to remove the residual fluorescence (71). Alkaline oxidation chemistry is used in a recently developed method called MxIF to chemically inactivate the fluorescent dyes after imaging (38, 73). CycIF combines oxidative inactivation and enzymatic antibody cleavage for sequential imaging (37). Multiplexed imaging can be also performed with indirect immunofluorescence, which does not require special antibody conjugation and allows amplification of signal from low-abundance markers using secondary antibodies (72). Experiments that rely on sequential staining and bleaching can take several days (37, 38, 72), tissue properties may change and sample handling can introduce error.

Second-generation sequential imaging approaches employ DNA-labeled antibodies (74, 75). Unlike methods that require time-consuming rounds of antibody staining, DNA-labeled antibodies are simultaneously applied to the specimen. The DNA oligonucleotides conjugated to the antibodies serve as barcodes that can be sequentially detected by fluorophore-labeled dNTPs in CODEX (75) or by fluorescent probes directly and indirectly linked to complementary DNA sequence in Exchange-PAINT (74) and immune-SABER (42). These approaches allow profiling of spatial signaling heterogeneity and reveal tissue organization-related network variations (38, 76). The capability for multiplexed super-resolution imaging in Exchange-PAINT enables the assessment of signaling protein interactions and clustering effects (77) but is time-consuming.

Challenges shared by all fluorescence-based methods are potential sample autofluorescence, which can be especially high in formalin-fixed, paraffin-embedded samples.

##### Mass Spectrometry-based Immunological Imaging Approaches

In imaging mass cytometry (IMC), all antibodies are applied simultaneously to stain tissue samples. A laser is then used to ablate antibody-stained samples spot by spot. A mixed argon and helium stream then transports the ablated materials into a mass cytometer. Proteins and protein modifications, such as phosphorylation, are quantified, preserving subcellular level (1 μm^2^) spatial information (Fig. 2, middle panel) (36, 78). IMC can be used to analyze proteins (including phosphoproteins) and RNAs simultaneously enabling, for example, analysis of correlations between transcriptional control and spatial signaling properties (79). Multiplexed ion beam imaging (MIBI), like IMC, uses metal-labeled antibodies for tissue staining. In MIBI, an oxygen duoplasmatron primary ion beam is used to liberate the antibodies to generate the secondary ion beam. Subsequently, a magnetic sector mass spectrometer or time-of-flight is used to detect the isotope abundances from the second ion beam from every pixel of analyzed sample (35, 80). The advantages of MIBI are that the same sample can be scanned multiple times and that the resolution can achieve 10 nm. The benefits of all mass spectrometry-based immunological imaging approaches are that samples can be stored indefinitely, that sample autofluorescence does not interfere with quantification, and that the dynamic range is orders of magnitude higher than in fluorescent-based approaches.

### Spatial 'Omics in Single-cell Analysis

#### 

##### MALDI-based Imaging

MALDI-based imaging mass spectrometry can be used to detect biomolecules, including lipids, metabolites, peptides, and proteins (81). Although MALDI-based imaging is mainly applied at tissue-level resolution, it has been used for unbiased quantitative and spatial profiling of the signal-mediating lipidome and metabolome (82) and in systemic assessments of disease states and drug responses (81, 83). A novel MALDI-based tissue imaging platform was recently developed that, because of optimized ionization efficiency, has a resolution at the subcellular level of 5 μm per pixel (84). Using a transmission geometry ion source, 1-μm resolution can be achieved with MALDI-based imaging systems, although at compromised sensitivity (85).

##### Spatial Transcriptomics

Several spatial transcriptomics approaches have been established based on various techniques, including fluorescent *in situ* sequencing (FISSEQ) (86), multiplexed MERFISH (41), and spatial barcoding (87). Data from these experiments can be used to infer signaling pathway activation and cell-to-cell communication. Spatial transcriptomics are also powerful methods for evaluating remote cell-signaling control mechanisms because mRNAs are used as expression readouts for secreted ligands (*e.g.* cytokines and chemokines) that are difficult to detect in proteome-based analyses (79).

### Live-cell Imaging

It is important to note that cell signaling transduction is a dynamic process that cannot be fully understood from snapshot measurements of transient network states. Information along the time dimension, in addition to the multiplexed signaling profiling, is therefore necessary to systematically decode the causality of signaling behaviors and to characterize network kinetics (88). As signaling events are mainly present intracellularly, they can be detected only after a fixation and permeabilization procedure that disrupts the signaling dynamics through time. Conventionally, serial snapshot information is acquired to enable the rebuilding of time dimension and the computational reconstruction of signaling trajectories (56, 89). Technically, these approaches do not fully resolve the transient events of signaling processing, and the computation inference becomes complicated when measured signaling behaviors that are highly heterogeneous. Several live-cell imaging methods exploit protein physical properties (*e.g.* kinase subcellular localization and protein proximity) to monitor signaling events through time (28, 90–95). Although these methods are not yet highly multiplexed, capturing information on central signaling nodes through time allows tracing the pathway and network behaviors.

#### 

##### Fluorescence Resonance Energy Transfer

Fluorescence resonance energy transfer (FRET) experiments are based on energy transfer between two proximate fluorophores that leads to a shift of the emission spectrum that is captured by microscopy. FRET can be used to monitor the proximity of interactive signaling proteins (95) or as a biosensor for phosphorylation events to indicate pathway activity in real-time (Fig. 2, right panel) (28, 94). FRET-based analysis characterizes single-cell temporal signaling states that can be correlated with functional readouts such as proliferation and differentiation. Given the broad fluorescent spectrum occupancy from each FRET sensor, multiplexing of FRET experiments to study complex signaling network behaviors is challenging. Several approaches to increase FRET multiplexing have been developed that rely on careful selection of fluorophores or image decoding and error propagation schemes. Up to six protein interaction/phosphorylation events have been measured simultaneously in a multiplexed FRET setup (96–98). FRET biosensors used in combination with a multi-parameter imaging platform have been used to separately monitor the activities of 40 signaling proteins in individual cells; the data generated were used to infer network dynamics comprehensively (92).

##### Activity-based Reporters

Many kinases, such as ERK, are translocated to the nucleus once activated. Thus, fluorescently-labeled versions of these proteins can be used to track signaling activities in real-time (90, 93, 99). Studies of kinase nuclear translocation at single-cell resolution revealed considerable heterogeneity in signaling dynamics (90) and noise-facilitated transcription output (93). A novel category of biosensors, known as kinase translocation reporters, was developed to convert phosphorylation into a nucleocytoplasmic shuttling event that allows monitoring of the activities of key signaling mediators including JNK, p38, and ERK simultaneously to identify temporal signaling crosstalk between the pathways (Fig. 2, right panel) (91). An important strength of these live-cell imaging technologies is the preservation of natural cellular states. The same imaged samples can be re-analyzed using other compatible single-cell methods. For instance, a study has coupled NFκB nuclear translocation analysis with single-cell RNA-sequencing to reveal three distinct cell subpopulations with different transcription profiles (29).

Each of the approaches discussed above has its advantages and limitations, as summarized in Table I. When selecting a single-cell method to study cell signaling networks, we suggest that experimentalists first accurately phrase their question and then assess whether it is necessary to acquire spatial or dynamics information, and then consider the factors of multiplexing, sensitivity, throughput, and cost.

## Computational Methods for Signaling Network Analysis Using Single-cell Information

Multiplexed measurements allow systematic assessment of network states and dynamics in one single experiment in which the multivariate dependences and high-dimensional distributions are precisely preserved. Network responses to perturbations can be visualized at the single-cell level using single-cell signaling fold changes (100), although the interpretation of signaling causality can be indirect. Recently developed computational approaches apply statistical inference to reconstruct signaling network structure (44, 54, 101, 102) and use mechanistic models to characterize network dynamics (103, 104).

For the reconstruction of signaling networks, Bayesian modeling has been applied in flow cytometry measurement of 11 intracellular phosphorylation sites with individual treatments of nine small-molecule inhibitors. Exploiting natural cellular variability and the re-shaping of multivariate distributions upon perturbations, a probabilistic network was assembled that replicates known pathway relationships and predicts novel network causalities (44). Alternatively, correlation-based statistics can be used to quantify relationships and dependences between measured parameters and are therefore widely used to assess the strength of signaling circuits and infer network structure and dynamics in both flow cytometry and transcriptomics data (105, 106).

In complex signaling regulatory networks, relationships between pairs of signaling proteins are often dependent on multiple parameters and non-monotonic in shape. Correlation analysis often fails to reflect the true strength of these relationships. Based on information theory, methods have been developed that use mutual information (MI) and maximal information coefficients (MIC) to quantify the relationships between two variables independently of their linearity and continuity (107, 108). A more advanced measure, termed DREMI, has been recently developed to quantify mutual information in a density-independent manner; this removes the bias of cell distribution. Networks reconstructed and quantified by DREMI recapitulate well-known signaling processes (54). In combination with experimental methods for tracing biological time during a cell transition (109), DREMI revealed signaling network reprogramming during cellular phenotypical shifts (55). Another density-independent measure, called binned pseudo-R^2^ (BP-R^2^), applies classical R^2^ statistics. The BP-R^2^ score reflects the strengths of signaling relationships in steady-state and dynamic studies with high accuracy (56).

Mechanistic models can reveal biochemical insights into a given signaling network and the functional heterogeneity within a cell population. Ordinary differential equations (ODEs) are commonly applied when mass action kinetics analyses are used to determine the concentration of signaling nodes over time. ODE models have been used to study network features such as feedback loops (110). A pilot single-cell analysis used ODE-constrained mixture modeling to study the variability of the response of phosphorylated ERK to stimulation with NGF in PC12 cells; two cell subpopulations with differential signaling responses caused by varied receptor abundance were identified (103). In another study, a hierarchal population model was developed, in combination with the single-cell modeling, to explain multiple levels of heterogeneity in NGF-treated PC12 cells (104).

## Accounting for Confounding Factors

Single-cell technologies have enabled characterization of differential signaling behaviors in cell populations that are masked by conventional batch measurements. However, these advantages also come with the challenge that multiple levels of confounding factors can bias the single-cell readouts (21, 111–117). Corrections for these potential confounding factors must be implemented in single-cell data analyses.

One of the most critical biological confounding factors is the cell cycle, as different signaling and transcriptional programs are active during each cell-cycle phase; these programs regulate events such as protein synthesis and DNA replication. Phosphorylation of signaling proteins is involved in cell-cycle regulation, and phosphoprotein levels vary through the cell-cycle progression (118, 119). For single-cell analysis, it is essential to distinguish variation because of cell-cycle stage from other sources of heterogeneity. Multiple computational methods are now available to account for cell-cycle effects in single-cell transcriptomic data, mass cytometry-based phosphorylation network analysis, and microscopic imaging analysis (21, 112, 114, 119).

As a signaling network is an integration of biochemical reactions, the rate of signal transduction is determined by the signaling protein concentration (103, 120). Protein concentration cannot be directly inferred from abundance measurements using the single-cell analysis techniques described here, as the volume is unknown. Studies have confirmed that cell size confounds single-cell measurements because size linearly correlates with most measured mRNA or protein or protein modification levels (21, 115). To account for the cell size, a method has been developed to experimentally estimate cell size based on total protein measurement. By normalizing the measured single-cell parameters to the cell size, relative concentration information can be gained (21).

The tissue dissociation protocol can also confound single-cell measurements. Both mechanical force and enzymatic treatment can trigger activation of stress signaling in cells, which will result in changes in the single-cell transcriptome and proteome (117). Minimizing the protocol length has been shown to reduce these potential artifacts (56). Alternatively, an *in situ* fixation approach can be used to minimize alterations in cellular phenotypes in tissue samples prior to analysis (121).

## Conclusion and Perspective

Signaling networks are centrally involved in information processing necessary for proper control of cell functions and cell fate. Deregulated signaling often leads to the emergence of disease. Recent advances in systems biology research have identified multiple layers of variability that contribute to heterogeneous signaling network states and dynamics. Importantly, the essential role of signaling network heterogeneity in the initiation and development of diseases, such as cancer, has been revealed. Many recently developed techniques are now capable of quantifying signaling events and network behaviors at the single-cell level.

Currently, up to 50 phosphorylation sites can be simultaneously quantified in mass cytometry-based single-cell proteomics analyses (50, 56). Imaging mass cytometry and several sequential imaging approaches provide spatial information in addition to signal profiling (36, 37, 74). These methods are ready to be used in systematic inference of signaling network behaviors in tissues at single-cell resolution (103, 104). Meanwhile, transcriptomics can be measured at the single-cell level to indicate activities of particular signaling pathways. Integrated with spatial information, transcriptomic methods have already furthered our understanding of paracrine signaling regulation, which involves secreted signaling proteins (40, 66, 86).

Technical advances have increased the multiplexity of antibody-based single-cell measurements. A caveat remains that suitable antibodies do not exist for many membrane-localized receptors or for many intracellular phosphorylation sites. Single-cell transcriptomic approaches can be used to assess mRNA expression globally and in an unbiased manner. Although these methods are prone to technical noise, making reliable detection of low-abundance mRNAs challenging, computational strategies have recently been described that mitigate this issue (122–125).

Integration of single-cell signaling characterization with multi-omics profiling will lead to an understanding of signaling circuits as well as feedback mechanisms between signaling pathways and transcriptional and epigenomic programs (79, 126, 127). Using oligonucleotide-tagged antibodies (57, 58), phosphorylation sites can be measured in combination with transcriptomic sequencing in the same cells. Spatial approaches, including imaging mass cytometry, now also allow simultaneous measurement of protein and RNA (79) and can be applied to answer questions regarding crosstalk between the regulators of the phosphoprotein network and transcription and the involvement of spatial factors, such as cell-to-cell contacts and protein localization, in such networks.
